# Effectiveness and Safety of Acupuncture Moxibustion Therapy Used in Breast Cancer-Related Lymphedema: A Systematic Review and Meta-Analysis

**DOI:** 10.1155/2020/3237451

**Published:** 2020-05-11

**Authors:** Huimin Jin, Yuying Xiang, Yuqian Feng, Yiting Zhang, Shan Liu, Shanming Ruan, Huamiao Zhou

**Affiliations:** ^1^The First Clinical Medical College of Zhejiang Chinese Medical University, Hangzhou, Zhejiang 310053, China; ^2^Center of Clinical Evaluation, Zhejiang Hospital of Traditional Chinese Medicine, Hangzhou, Zhejiang 310006, China; ^3^Department of Medical Oncology, The First Affiliated Hospital of Zhejiang Chinese Medical University, Hangzhou, Zhejiang 310006, China

## Abstract

**Objective:**

To evaluate the effectiveness and safety of acupuncture moxibustion therapy (AMT) for the breast cancer-related lymphedema (BCRL).

**Methods:**

Four English databases (MEDLINE, PubMed, Embase, and Cochrane CENTRAL) and four Chinese databases were searched from their inception to Feb 1, 2020. Eligible randomized controlled trials (RCTs) investigating AMT against any type of controlled intervention in patients for BCRL and assessing clinically relevant outcomes (total effective rate, circumference difference, and Karnofsky performance score) were included. The methodological quality of all selected trials was estimated in accordance with the guidelines published by the Cochrane Collaboration. Review Manager 5.3 was used to conduct analyses.

**Results:**

Twelve eligible RCTs are confirmed. Most of the trials selected are regarded as low methodological quality. Compared with Western medicine, physiotherapy, and functional training, traditional AMT has significantly higher treatment effect (RR 1.03 (95% CI: 1.22, 1.45); *p* < 0.00001). In comparison with physiotherapy, AMT is better in reducing edema symptoms (MD = −0.77; 95% CI (−1.13–0.41); *p* < 0.00001). Moreover, pooled results demonstrate that AMT results in better outcomes than functional training and Western medicine in improving Karnofsky performance score of BCRL patients (SMD = 0.69; 95% CI (0.38–1.00); *p* < 0.00001).

**Conclusion:**

This systematic review and meta-analysis provides evidence that AMT is serviceable and safe in treating BCRL. With the limited number of available studies and methodology drawbacks, further high-quality RCTs with reasonable designs are still warranted.

## 1. Introduction

Worldwide, breast cancer is the most common cancer and the second leading cause of cancer-related deaths in females [1]. With the increase of the cure rate and survival rate of breast cancer patients, the postoperative quality of life of breast cancer patients has attracted more and more attention. Breast cancer-related lymphedema (BCRL) is recognized as one of the most serious complications of breast cancer surgery [2]. Study [3] shows that the 10-year cumulative incidence of BCRL is 41.1% in women that underwent ALND as part of the surgical treatment for breast cancer. Patients with BCRL suffer from weight gain, skin thickening, arm swelling, and reduced shoulder range of motion, resulting in restrictions on daily activities, as well as pain, anxiety, and depression [4]. BCRL is caused by the accumulation of lymph fluid in tissues due to surgery, radiotherapy, or other reasons that disturb the transport ability of the lymphatic system [5]. Popular methods of treating BCRL adopted by modern medicine are complex decongestive therapy (CDT), drug intervention (diuretic or dehydration), and surgical reconstruction of lymphatic vessels [6, 7]. However, these methods cannot fundamentally tackle the problem. How to treat BCRL effectively is still a clinical challenge.

Traditional Chinese medicine (TCM) approaches include the use of herbal medicines, acupuncture moxibustion therapy (AMT), dietary therapy, and tai chi/qigong. In TCM theory, human bodily functions are controlled by the “meridian” and “Qi and blood” systems [8]. There are 365 designated acupoints located along 14 meridians which can be used to stimulate, balance, and harmonize the yin and yang by relieving blockages in the flow of Qi. Acupoint stimulation is a complex, ritualistic somatosensory intervention with multiple components. This method of healing has been used to promote homeostasis of the body's organs. Although AMT is a component of TCM that may be traced back to >2,500 years in China, it is becoming a popular complementary and alternative treatment in Western countries and is growing in popularity worldwide. According to the theory of TCM [9–11], upper limb lymphedema is closely related to san-yang meridian and san-yin meridian. Stimulating the meridian system of patients makes the blocked lymphatics flow, helps the striated muscles contact, and promotes the backflow of lymph to relieve the pain of lymphedema. In order to clarify the therapeutic effect of AMT on BCRL, domestic and foreign researchers have conducted relevant randomized controlled trials (RCTs) [12–15], but the sizes of samples considered by former researches are small, and the quality of these studies varies. Therefore, systematic review and meta-analysis are applied in this study to evaluate the effectiveness and safety of AMT in treating BCRL, so as to provide support and reference for clinical practice.

## 2. Methods

The meta-analysis was performed in accordance with the Preferred Reporting Items for Systematic Reviews and Meta-Analyses (PRISMA) guidelines [16].

### 2.1. Literature Search Strategy

Electronic databases including Chinese Biomedical Literature Database (CBM), Chongqing VIP Information (VIP), China National Knowledge Infrastructure (CNKI), Wanfang Data (Wanfang), MEDLINE, PubMed, Embase, and Cochrane CENTRAL were searched systematically in English and Chinese between their inception and Feb 1, 2020 to identify eligible studies. Reference lists of relevant articles are also hand-searched. A grey literature search was performed through Google Scholar to capture dissertations, theses, and conference proceedings that met the inclusion criteria [17]. The search strategy adopted the combination of the controlled vocabulary (Emtree term and MeSH term) and free-text term. The key words including “breast cancer related lymphedema” and “acupuncture and moxibustion” were considered for databases searching, and relevant derivatives were used wherever appropriate. The complete search strategy for each database is available in Supplementary Material 1 (available here).

### 2.2. Study Selection

Eligible studies would be selected if the following criteria were met.

#### 2.2.1. Participant

Only female, adult patients (aged ≥ 18 years) with lymphedema caused by surgery, radiation, and/or chemotherapy for breast cancer were included. The arm circumference was measured at the midpoint of both upper limbs of the patient. When the affected side circumference was 2 cm larger than that of the unaffected side, it was diagnosed as lymphedema.

#### 2.2.2. Intervention

Patients in the treatment group conducted acupuncture moxibustion therapy (no restrictions on acupoint selection, operation method, and course of treatment) alone or combined with other Western medicine, routine functional training, and physiotherapy. Also, patients in the control group conducted nonacupuncture therapy, including Western medicine, functional training, and physiotherapy.

#### 2.2.3. Comparator

Trials comparing AMT to without AMT (Western medicines or physical therapy).

#### 2.2.4. Outcome

(1) Total effective rate: the total effective rate could be calculated in two ways. (I) Effective index (%) = (pretreatment circumference of the affected arm − posttreatment circumference of the affected arm)/(pretreatment circumference of the affected arm − pretreatment circumference of the unaffected arm); significantly effective: effective index > 90% or above; effective; 10–90%; ineffective <10%. Significantly effective + effective = total effective rate. (II) The reduction in the difference between circumferential diameters of the affected side and the contralateral side of the elbow joint after treatment compared with that before treatment—excellent: reduction of 75% or above; good: reduction of 25–50%; and ineffective: reduction of less than 25%. Excellent + good + effective = total effective rate. (2) Circumference difference. (3) Karnofsky performance score (KPS).

#### 2.2.5. Study design

Randomized controlled trials.

Additionally, the reasons for excluding the studies were as follows:

- Patients with recurrent or remote metastatic
- Non-RCTs, meta-analysis, reviews, case reports, and involved animal or cell experiments
- Full text not available

Study selection process was independently conducted by two of the authors (J.-H.M. and X.-Y.Y.) who received systematic training, respectively. Titles and abstracts of publications were read for the initial selection. If an article cannot be determined whether it is qualified or not, a full-text review version was necessary. Any disagreements during the study selection were resolved by an additional discussion with a third reviewer (F.-Y.Q.).

### 2.3. Data Collection

In the current systematic review and meta-analysis, data were extracted from the selected articles by two researchers (J.-H.M. and X.-Y.Y.) independently to ensure transparency and discrepancies on data collection. A predefined data extraction sheet was designed to collect the raw data. The recorded data included basic characteristics of studies, intervention measures, adverse events, outcome indicators, and some details about the treatment group. Disagreements were discussed by the two reviewers to reach a consensus; if disagreement was still unresolved, a third reviewer (F.-Y.Q.) was consulted.

### 2.4. Quality Assessment

The methodological quality of included RCTs was measured by two reviewers (J.-H.M. and X.-Y.Y.) with the Cochrane risk of bias assessment tool, which is recommended in the Cochrane Handbook of Systematic Reviews of Interventions. The detailed list of quality items included the following criteria: method of randomization; allocation concealment; whether the researcher and the participants are blind; blind evaluation of outcome; integrity of outcome data; and selective reporting bias and other potential biases. Each item was classified into three levels, i.e., low risk, high risk, and unclear risk for insufficient information. Disagreements in the assessment process were resolved by additional discussions with a third researcher (F.-Y.Q.) to reach consensus.

### 2.5. Grading the Quality of Evidence

Besides, the quality of evidence for main outcomes was evaluated by two reviewers (J.-H.M. and X.-Y.Y.) independently through GRADE Pro 3.6 software. Disagreement on quality assessment was resolved by discussion. The Grading of Recommendations Assessment, Development, and Evaluation (GRADE) system classifies the quality of evidence as 4 levels: high quality (㊉ ㊉ ㊉ ㊉), moderate quality (㊉ ㊉ ㊉), low quality (㊉ ㊉), and very low quality (㊉) [18].

### 2.6. Statistical Analysis

This study analyzed the collected data using RevMan software (version 5.3; Cochrane Collaboration). For dichotomous data, we will estimate the effect size using risk ratios (RRs). For continuous data, if the outcomes had been measured with the same unit, the results were calculated as mean differences (MD), otherwise, calculated as standardized mean difference (SMD). The confidence interval (CI) is set as 95%. *I*^2^ statistic was conducted to investigate the interstudy heterogeneity (*I*^2^ = 0%, no heterogeneity; 0% < *I*^2^ ≤ 25%, insignificant heterogeneity; 25% < *I*^2^ ≤ 50%, moderate heterogeneity; 50% < *I*^2^ ≤ 75%, high heterogeneity; and *I*^2^ > 75%, extremely high heterogeneity). Given clinical heterogeneity, subgroup analysis was necessary. When heterogeneity was too large, descriptive statement was used [19]. As recommended by the Cochrane Handbook for Systematic Reviews of Interventions [20], publication bias was evaluated by funnel plots when the number of included studies was greater than or equal to 10. A fixed-effect (FE) model was performed to estimate a combined RR. If significant heterogeneity was detected among trials, a random-effect (RE) model would be applied. Sensitivity analysis will be conducted to identify the influence of single study on the synthesized results.

## 3. Results

### 3.1. Search Results

**Figure 1:**
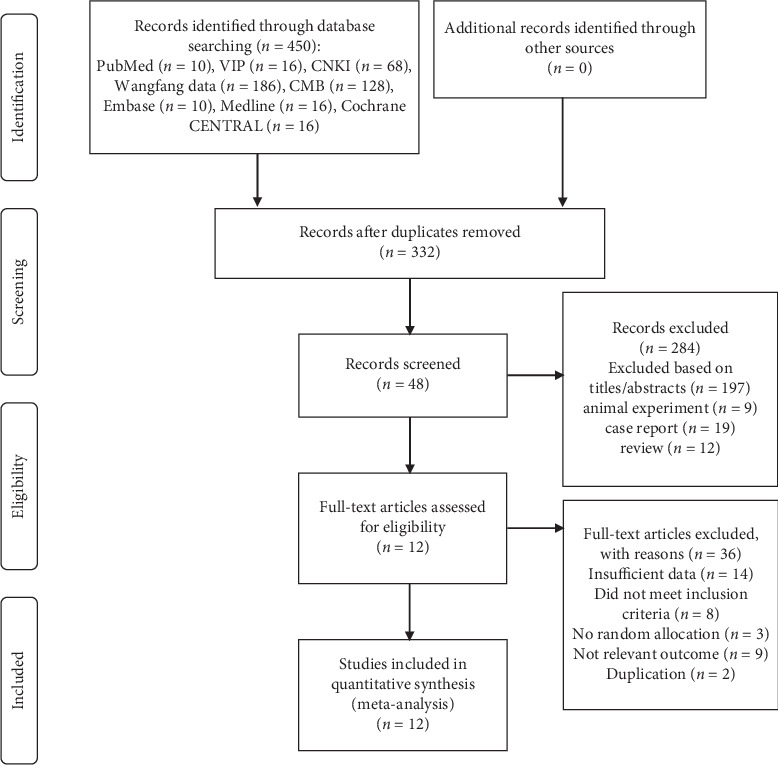
Flow diagram of the study selection process.

A total of 450 articles were retrieved from PubMed, Embase, MEDLINE, and Cochrane CENTRAL, etc. After removing the duplicates and an initial review, 48 potentially useful relevant references remained for further assessment. The full texts of the 48 articles were downloaded and evaluated in detail for qualification. Due to various reasons, 36 articles of them were excluded. Meanwhile, no article was identified through the manual search of the reference lists related. Finally, twelve RCTs [21–32] were selected in this meta-analysis for the assessment of efficiency and safety of AMT.  Figure 1 presents the specific process of literature selection based on PRISMA guidelines.

### 3.2. Study Characteristics

Twelve studies with 778 participants were taken into consideration in the final analysis. Two studies were published in English, and the other ten studies were published in Chinese. All included studies were RCTs and adopted a two‐armed parallel group design. These articles were published between 2012 and 2020, and the sample sizes ranged from 30 to 80. Patients included in all studies met the criteria of BCRL. We analyzed the therapeutic outcomes of AMT with or without physical therapy or functional exercise. Moxibustion therapy was used in 7 studies [23–29], and manual acupuncture (MA) was used in 2 studies [22, 30] for the treatment of BCRL. One study [21] investigated the effect of electroacupuncture versus manual acupuncture and physical therapy simultaneously. Besides, two studies [31, 32] employed cupping and acupressure, respectively, to evaluate the effect of AMT. The general characteristics of each identified study are summarized in Table 1, and the specific interventions of each study are shown in Table 2.

### 3.3. Risk of Bias Assessment

**Figure 2:**
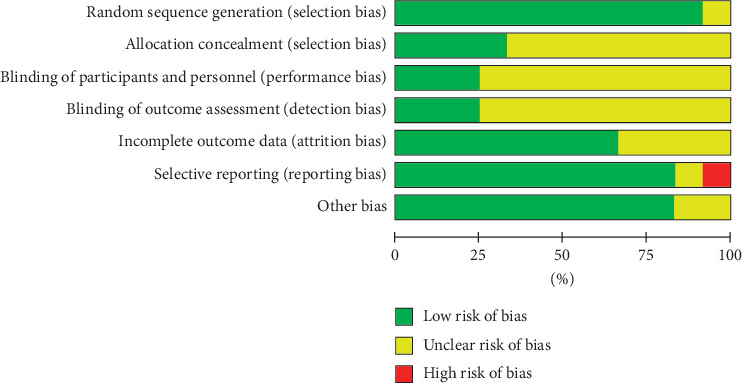
Risk of bias graph.

The results of the quality evaluation of the twelve studies are described in Figure 2. For most of the studied articles, the methodological information was incomplete. Participants in all trials were randomly divided into treatment or control groups, and the two trials did not indicate the method of random sequence generation clearly [29, 33]. Only one study [30] illustrated the method of allocation concealment in detail, and none of the studies mentioned adequate blinding of patients and the doctors. Evidently, one of the studies [23] had a high selective reporting bias, because it failed to report all predefined outcomes in the results. Furthermore, it was hard to estimate whether there was other bias in all the eligible trials included.

### 3.4. Effects of the Interventions

#### 3.4.1. Total Effective Rate

**Figure 3:**
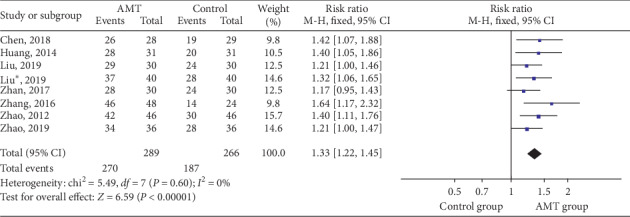
Forest plot of dichotomous data outcomes: total effective rate.

The total effective rate was reported in eight studies [21–23, 25–27, 31, 32] with 289 participants in experimental and 266 in control groups to evaluate the curative effect of AMT. Compared to Western medicine, physiotherapy, and functional training, traditional AMT had significantly better total effective rate (RR 1.03 (95% CI: 1.22, 1.45); *p* < 0.00001), which is shown in Figure 3.

**Figure 4:**
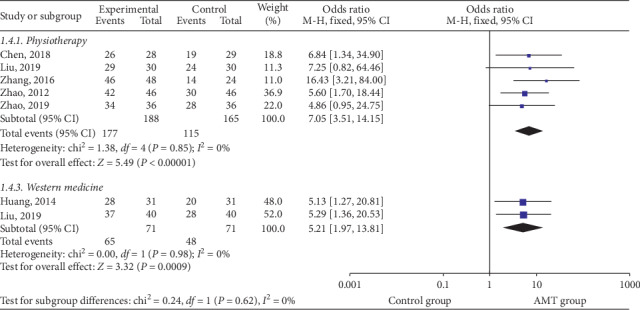
Subgroup analysis was performed based on different intervention options.

Given the clinical heterogeneity, subgroup analysis was performed based on different intervention options [22, 24, 26–28, 32]. The result of subgroup analysis is shown in Figure 4.

#### 3.4.2. Circumference Difference

**Figure 5:**
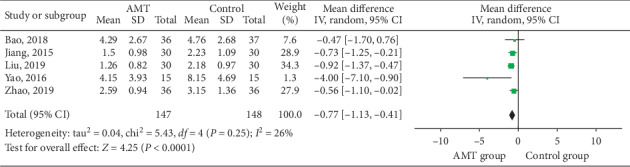
Forest plot of continuous data outcomes: circumference difference.

In five trials [21, 26, 28–30], the circumference difference of the midpoint of the upper arm between the affected side and the healthy side before and after treatment was used as the main outcome index. The effect of AMT was indirectly evaluated by the change of swelling degree. The meta-analysis of these trials demonstrated that AMT was better than physiotherapy (MD = −0.77; 95% CI (−1.13–0.41); *p* < 0.00001), which is shown in Figure 5.

#### 3.4.3. Karnofsky Performance Score (KPS)

**Figure 6:**
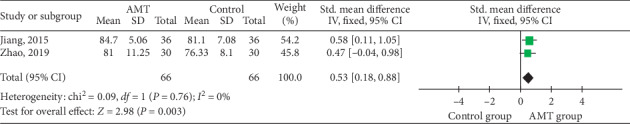
Meta-analysis of the RCTs comparing living quality between AMT group and control group.

The KPS score was directly proportional to the health status of the patients and was analyzed in two RCTs [21, 28] to evaluate the living quality of participants. The results revealed a better effect on the KPS score in the AMT group compared with the control group (SMD = 0.53; 95% CI (0.18–0.88); *p* < 0.00001), which is shown in Figure 6.

### 3.5. Adverse Events

Five of the studied trials mentioned adverse effects [21, 24, 29, 30, 32], but only one trial [24] reported that a participant developed local skin flushing accompanied by itching after moxibustion. The remaining four trials presented no adverse events, and the other seven trials did not report any information on adverse effects.

### 3.6. Sensitivity Analysis

To assess the robustness of overall results of the meta-analysis, we performed a sensitivity analysis by removing each trial individually. The result showed that the results were not modified after exclusion of any included trials.

## 4. Discussion

### 4.1. Overview of Findings

The goal of this study was to evaluate the effectiveness and safety of AMT in patients with BCRL. After literature screening, twelve RCTs with 778 participants were identified for further analysis. On account of the evidence we assessed, the conclusion that can be reached is that AMT could be considered as an alternative option to enhance the efficacy of BCRL treatment. Eight trials showed that AMT combined with physiotherapy or functional exercise for the treatment of BCRL significantly improved the total effective rate [21–23, 25–27, 31, 32], in comparison with using conservative treatment alone. Based on changes in the difference of upper limbs before and after treatment, five trials suggested that AMT or AMT plus physical therapy was more effective than physical therapy alone [21, 26, 28–30]. The differences in arm dimension changes were smaller in patients treated with AMT than those in untreated patients. These results showed that acupuncture and moxibustion enhance the beneficial effects of physical therapy. Moreover, pooled data from five studies showed that AMT (including cupping) had advantage over Western medicine and physiotherapy at improving the quality of life among BCRL patients [21, 24, 25, 28, 32]. Among the included studies, only one trial clearly recorded the adverse events that occurred during the treatment period [24]. It can be said that AMT is basically safe and hardly bring about harm to the human body. Several studies have taken moxibustion as an intervention measure [23–29]. The results indicated that moxibustion efficiently alleviated the symptoms of edema in BCRL patients, without any obvious adverse events. Hence, after several times of professional guidance, patients could carry out moxibustion by themselves, which is free of time and place restrictions. These findings indicated that the efficacy of AMT was higher compared to other conservative treatments. Apart from this, since the adverse events of AMT were negligible, it was extraordinary attractive to women with BCRL.

### 4.2. Possible Rationale of AMT for BCRL

Lymphedema is a potential adverse effect caused by surgery or radiotherapy injury, which is defined as soft tissues swelling as a result of accumulation of protein-rich interstitial fluid [34, 35]. Breast cancer-related lymphedema (BCRL) is a chronic disease that can lead to edema, hypertrophy, and even fibrosis of the upper extremity. According to the theory of traditional Chinese medicine (TCM), the main reason of BCRL is that after surgery or radiotherapy of breast cancer, the local meridians and collaterals of the upper limbs are blocked, which affects the circulation of Qi and blood, leading to the stagnation and accumulation of body fluids under the local skin [9]. The statistical results show that AMT has a positive effect on relieving upper extremity edema, which is often occurred after the radical mastectomy. Both of acupuncture and moxibustion are the treasures of Chinese medicine culture and have significant clinical effects [10, 11]. According to the theory of traditional Chinese medicine, the acupuncture moxibustion therapy (AMT) can dredge meridians and regulate the circulation of Qi and blood by stimulating acupoints [8]. The stimulation of acupoint not only improves the blood circulation locally, but also regulates the function of the whole body to build up the immune system [36–38]. Modern medicine demonstrates that the effects of physiology acupuncture might involve with the peripheral nervous system (PNS), through reducing the production of free radical to promote the release of anti-inflammatory factors [39–41].

### 4.3. Implication from This Research

In future studies, more clinical trials with rigorous designs should be conducted to explore the efficiency and safety of AMT on BCRL treatment. Explicit random method, adequate allocation concealment, and blind design are requisite, but rarely utilized in the studied researches. Meanwhile, if AMT has a long-term safety effect on the treatment for lymphedema, the follow-up time should be prolonged and the adverse events occurred during the period are supposed to be itemized to ensure the correctness of the researches. Besides, the standardized dedicated scales can be utilized to effectively evaluate the improvement of physical activity of patients. Last, which specific program and which acupoints have the best effect on reducing BCRL is one of the directions that should be explored in the future.

### 4.4. Limitation

Allowedly, limitations of this study should be taken into consideration. First, although the related researches have been collected as many as possible, the sample size of this studies included is limited, which reduces the accuracy of the analysis and only provides a little clinical evidence. Second, most of the RCTs selected are conducted and published in China, which may lead to regional bias. AMT originated in ancient China, the interest of Western society in AMT has developed in recent decades. However, in terms of the number of studies on acupuncture treatment, China is far higher than the Western countries. Third, BCRL has the characteristics of long-term and recurrent episodes, but the duration of the treatment and the follow-up period are too short to evaluate the long-term efficacy. Last, there are different examinations that are used for the clinical evaluation of the severity of BCRL, such as near-infrared fluorescence (NIRF), indocyanine green (ICG) lymphography, MR lymphography, and photoacoustic imaging [42]. However, within the study we included, upper arm circumference was used considering the fact that it is the most intuitive and measurable data. Due to factors such as the body shape of patients and measurement time, the deviations in the actual measurement are inevitable.

## 5. Conclusion

To summarize, the presented meta-analysis provides evidence that AMT, known as an intervention, could be an effectual option in the treatment of BCRL. However, the conclusion should be interpreted with some caution due to the limitations associated with this systematic review and meta-analysis. In the future, more well-designed, high-quality, multicenter, large-sample randomized trials with long-term follow-up will be required to confirm current results.

## Figures and Tables

**Table 1:** Characteristics of included studies.

Study ID	Number (T/C)	Age, years (T/C)	Interventions				^#^Outcomes	Quality of the evidence (GRADE)
			Control group	Treatment group	Follow-up visit	Duration		
Zhao, 2019	36/36	43.25 ± 9.29/46.25 ± 9.72	Physiotherapy	Physiotherapy + electroacupuncture + auricular acupuncture	—	2 months	1, 2, 4	㊉ ㊉ ㊉Moderate
Zhan, 2017	30/30	30–70, mean: 57.84 ± 4.48	Functional training	Functional training + manual acupuncture	—	28 days	1	㊉ ㊉ ㊉Moderate
Zhao, 2012	46/46	47.2/46.4	Physiotherapy	Moxibustion	4 weeks	9 weeks	1	㊉ ㊉ ㊉Moderate
Yang, 2017	22/23	58.25 ± 6.19/59.42 ± 7.02	Physiotherapy	Moxibustion	4 months	4 weeks	3, 4	㊉ ㊉ ㊉Moderate
Liu^*∗*^, 2019	40/40	46.37 ± 14.53/49.32 ± 13.22	Western medicine	Moxibustion	—	28 days	1, 3	㊉ ㊉ ㊉Moderate
Liu, 2019	30/30	55 ± 11/55 ± 10	Physiotherapy	Moxibustion	—	42 days	1, 2	㊉ ㊉ ㊉Moderate
Huang, 2014	31/31	30–73, mean: 45.5	Functional training + Western medicine	Functional training + moxibustion	—	6 weeks	1	㊉ ㊉ ㊉Moderate
Jiang, 2015	30/30	18–70	Physiotherapy	Physiotherapy + moxibustion	—	8 weeks	2	㊉ ㊉ ㊉Moderate
Yao, 2016	15/15	56.2 ± 5.82/55.8 ± 5.02	Physiotherapy	Moxibustion	—	30 days	2, 4	㊉ ㊉ ㊉ ㊉High
Bao, 2018	40/42	65 (54, 71)/58 (49, 70)	Physiotherapy	Manual acupuncture	3 months	6 weeks	2, 4	㊉ ㊉ ㊉ ㊉High
Chen, 2018	30/30	55 ± 11.8/54.93 ± 9.5	Physiotherapy	Acupressure + Chinese medicine packets	—	28 days	1	㊉ ㊉ ㊉Moderate
Zhang, 2016	50/25	59.90 ± 7.02/56.96 ± 5.33	Functional training	Functional training + bloodletting puncture and cupping	—	50 days	1, 3, 4	㊉ ㊉ ㊉Moderate

^#^Outcomes: (1) total effective rate; (2) circumference difference; (3) KPS score; and (4) adverse effects.

**Table 2:** Specific interventions of included studies.

Study ID	Interventions		
	Control group	Treatment group	Acupuncture moxibustion course
Zhao, 2019	Air pressure treatment	Electroacupuncture at LI4, LI11, SJ5, LI15, RN6, ST25, RN12, SP10, ST36, RN9, SP9, CO17, CO18, TG2p + air pressure treatment	3 × per week for 2 months (24 total)
Zhan, 2017	Functional training (move up with hands against the wall, rotation and extension of upper limbs, and pulling a rope)	Acupuncture at RN12, RN10, RN6, RN4, ST24, ST26	1 × per day for 28 days (28 total)
Zhao, 2012	Comprehensive conservative treatment measures (raising the affected limb, hot compress, physical therapy, centripetal massage, wearing tights, using elastic bandage)	Moxibustion at LI4, LI15, SJ5, LI11, GB21, SI9, SJ14, LI14, LU1, LU7, RN7, SP9, ST36, LR3	5 × per week for 9 weeks (45 total)
Yang, 2017	Pneumatic circulation-driven pressure therapy	Moxibustion at LI11, LI14, SI9, DU3	2 × per week for 4 weeks (8 total)
Liu^*∗*^, 2019	Oral diosmin	Moxibustion at LI15, SJ5, SP9, LI11, RN9, ST36	1 × 2 days for 28 days (14 total)
Liu Y., 2019	Manual lymphatic drainage	Warm needling at LI15, GB21, SJ14, LI4, SJ5, LI11, LU7, RN9, SP9	1 × per day for 42 days (42 total)
Huang, 2014	Functional training + life care + oral hydrochlorothiazide, spironolactone	Moxibustion at LI15, GB21, SJ14, LI4, SJ5, LI11, LU7, RN9, SP9, LI14, SI9	5 × per week for 6 weeks (30 total)
Jiang, 2015	Air pressure wave therapy	Thunder‐fire moxibustion at ST36, LI4, LI11, DU14, GB21, LR3, BL17, RN6	5 × per week for 8 weeks (40 total)
Yao, 2016	Oral diosmin	Acupuncture at LI15, SJ5, SJ14 and moxibustion at SJ5, LI15, and SJ14	1 × 2 days for 30 days (15 total)
Bao, 2018	Standard lymphedema treatments, such as exercise and compression garments	Acupuncture at CV12, CV3, TE14, LI15, LU5, LI4, ST36, SP6	2 × per week for 6 weeks (12 total)
Chen, 2018	Functional training + life care	Chinese medicine packet plus acupoint massage at LI1, SJ5, LU5, LU2, GB21, BL13	1 × per day for 28 days (28 total)
Zhang, 2016	Upper limbs functional exercise	Cupping at the most swollen site after bloodletting with a plum blossom needle	1 × 5 days for 50 days (10 total)

## Supplementary Materials

Supplementary Materials For the complete search strategies used in each database, refer to Supplementary Material 1.
