# Supplementary material for: Effectiveness and Safety of Acupuncture Moxibustion Therapy Used in Breast Cancer-Related Lymphedema: A Systematic Review and Meta-Analysis
Source: Evid Based Complement Alternat Med. 2020 May 11;2020:3237451. doi: 10.1155/2020/3237451 (PMC7240793; doi:10.1155/2020/3237451)
Supplement: Supplementary Materials — For the complete search strategies used in each database, refer to Supplementary Material 1. [file 3237451.f1.docx]

Search Strategy

| Database | # | Search syntax | Citations |
| --- | --- | --- | --- |
| 1)  Embase | 1 | “breast cancer-related lymphedema”/exp |  |
|  | 2 | (“Breast Cancer Lymphedema” OR “Breast Cancer Lymphedema” OR “Breast Cancer Lymphedemas” OR “Lymphedema, Breast Cancer” OR “Breast Cancer Treatment-Related Lymphedema” OR “Lymphedema, Postmastectomy” OR “Breast Cancer-Related Arm Lymphedema” OR “Breast Cancer Related Arm Lymphedema” OR “Breast Cancer Related Lymphedema” OR “Postmastectomy Lymphedema” OR “Breast Cancer Treatment Related Lymphedema” OR “Lymphedemas, Postmastectomy” OR “Postmastectomy Lymphedemas” OR “Post-mastectomy Lymphedema” OR “Post-mastectomy Lymphedemas”):ti,ab,kw,de |  |
|  | 3 | (acupuncture OR acupress OR acupoint OR electroacupuncture OR "Zhen Jiu" OR ZhenJiu OR meridian OR "Ching Lo" OR Chinglo OR "Jing Luo" OR Jingluo OR moxibustion OR auriculotherapy OR TENS OR PENS OR ((transcutaneous OR percutaneous OR transdermal OR cutaneous) NEAR/3 (stimulat* OR electrostimulat* OR neurostimulat*))):ti,ab,kw,de |  |
|  | 4 | acupuncture/exp OR "transcutaneous electrical nerve stimulation"/exp OR moxibustion/exp |  |
|  | 5 | (#1 OR #2) AND (#3 OR #4) | **13** |
|  | 6 | #5 AND (“crossover procedure”:de OR “double-blind procedure”:de OR “randomized controlled trial”:de OR “single-blind procedure”:de OR (random* OR factorial* OR crossover* OR cross NEXT/1 over* OR placebo* OR doubl* NEAR/1 blind* OR singl* NEAR/1 blind* OR assign* OR allocat* OR volunteer*):de,ab,ti) | **10** |
| 2)  Medline | 1 | (lymph*edem* OR (lymph* ADJ3 (edem* OR oedem*)) OR (milroy* ADJ3 disease) OR elephantias*s).mp |  |
|  | 2 | exp Lymphedema/ |  |
|  | 3 | (acupunctur* OR acupress* OR acupoint* OR electroacupunctur* OR "Zhen Jiu" OR ZhenJiu OR meridian* OR "Ching Lo" OR Chinglo OR "Jing Luo" OR Jingluo OR moxibustion* OR auriculotherapy OR TENS OR PENS OR ((transcutaneous OR percutaneous OR transdermal OR cutaneous) ADJ4 (stimulat* OR electrostimulat* OR neurostimulat*))).mp |  |
|  | 4 | exp "Acupuncture"/ OR exp "Acupuncture Therapy"/ OR exp "Acupressure"/ OR exp "Transcutaneous Electric Nerve Stimulation"/ |  |
|  | 5 | (#1 OR #2) AND (#3 OR #4) | **35** |
|  | 6 | #5 AND (randomized controlled trial.pt. OR controlled clinical trial.pt. OR randomi*ed.ab. OR placebo.ab. OR drug therapy.fs. OR randomly.ab. OR trial.OR. or groups.ab. NOT (exp animals/ NOT humans.sh.)) | **16** |
| 3)  Cochrane  CENTRAL | 1 | MeSH descriptor: [Breast Cancer Lymphedema] explode all trees |  |
|  | 2 | (“Breast Cancer Lymphedema” OR “Breast Cancer Lymphedema” OR “Breast Cancer Lymphedemas” OR “Lymphedema, Breast Cancer” OR “Breast Cancer Treatment-Related Lymphedema” OR “Breast Cancer Treatment Related Lymphedema” OR “Breast Cancer-Related Arm Lymphedema” OR “Breast Cancer Related Arm Lymphedema” OR “Breast Cancer Related Lymphedema” OR “Postmastectomy Lymphedema” OR “Lymphedema, Postmastectomy” OR “Lymphedemas, Postmastectomy” OR “Postmastectomy Lymphedemas” OR “Post-mastectomy Lymphedema” OR “Post-mastectomy Lymphedemas”):ti,ab,kw |  |
|  | 3 | MeSH descriptor: [Acupuncture Therapy] explode all trees |  |
|  | 4 | MeSH descriptor: [Moxibustion] explode all trees |  |
|  | 5 | (“Acupuncture Therapy” OR “Acupuncture Treatment” OR “Acupuncture Treatments” OR “Treatment, Acupuncture” OR “Therapy, Acupuncture” OR “Pharmacoacupuncture Treatment” OR “Treatment, Pharmacoacupuncture” OR “Pharmacoacupuncture Therapy” OR “Therapy, Pharmacoacupuncture” OR Acupotomy OR Acupotomies OR Needling OR “dry needling” OR “needling therapy” OR “Warm needle acupuncture” OR Electroacupuncture OR moxibustion OR moxa OR “moxa cone” OR “moxa stick”):ti,ab,kw |  |
|  | 6 | (#1 OR #2) AND (#3 OR #4 OR #5) | **16** |
| 4)  PubMed | 1 | "Breast Cancer Lymphedema"[Mesh] |  |
|  | 2 | (Breast Cancer Lymphedema[Title/Abstract]) OR (Breast Cancer Lymphedema[Title/Abstract]) OR (Breast Cancer Lymphedemas[Title/Abstract]) OR (Lymphedema, Breast Cancer[Title/Abstract]) OR (Breast Cancer Treatment-Related Lymphedema[Title/Abstract]) OR (Breast Cancer Treatment Related Lymphedema[Title/Abstract]) OR (Breast Cancer-Related Arm Lymphedema[Title/Abstract]) OR (Breast Cancer Related Arm Lymphedema[Title/Abstract]) OR (Breast Cancer Related Lymphedema[Title/Abstract]) OR (Postmastectomy Lymphedema[Title/Abstract]) OR (Lymphedema, Postmastectomy[Title/Abstract]) OR (Lymphedemas, Postmastectomy[Title/Abstract]) OR (Postmastectomy Lymphedemas[Title/Abstract]) OR (Post-mastectomy Lymphedema[Title/Abstract]) OR (Post-mastectomy Lymphedemas[Title/Abstract]) |  |
|  | 3 | "Acupuncture Therapy"[Mesh] |  |
|  | 4 | "Moxibustion"[Mesh] |  |
|  | 5 | (Acupuncture Therapy[Title/Abstract]) OR (Acupuncture Treatment[Title/Abstract]) OR (Acupuncture Treatments[Title/Abstract]) OR (Treatment, Acupuncture[Title/Abstract]) OR (Therapy, Acupuncture[Title/Abstract]) OR (Pharmacoacupuncture Treatment[Title/Abstract]) OR (Treatment, Pharmacoacupuncture[Title/Abstract]) OR (Pharmacoacupuncture Therapy[Title/Abstract]) OR (Therapy, Pharmacoacupuncture[Title/Abstract]) OR (Acupotomy[Title/Abstract]) OR (Acupotomies[Title/Abstract]) OR (Needling[Title/Abstract]) OR (dry needling[Title/Abstract]) OR (needling therapy[Title/Abstract]) OR (Warm needle acupuncture[Title/Abstract]) OR (Electroacupuncture[Title/Abstract]) OR (moxibustion[Title/Abstract]) OR (moxa[Title/Abstract]) OR (moxabustion[Title/Abstract]) OR (moxa cone[Title/Abstract]) OR (moxa stick[Title/Abstract]) |  |
|  | 6 | (#1 OR #2) AND (#3 OR #4 OR #5) | **22** |
|  | 7 | #6 AND ((Randomized controlled trial[Publication Type]) OR (Controlled clinical trial[Publication Type]) OR (randomi*ed[Title/Abstract]) OR (placebo[Title/Abstract]) OR (randomly[Title/Abstract]) OR (trial[Title/Abstract]) OR (groups[Title/Abstract])) | **10** |
| 5)  CNKI | 1 | SU=(乳腺癌+乳癌+乳腺恶性肿瘤)*(水肿+上肢淋巴水肿+淋巴水肿+肿胀)*(临床观察+随机+临床研究+对照组+实验组+治疗组)*(针灸+针刺+艾灸+穴位+针+灸+穴位注射+电针+体针) | **68** |
| 6)  WangFang Data | 1 | 主题:(乳腺癌+乳癌+乳腺恶性肿瘤)*(水肿+上肢淋巴水肿+淋巴水肿)*(临床观察+随机+临床研究+对照组+实验组+治疗组)*(针灸+针刺+艾灸+穴位+针+灸+穴位注射+电针+体针) | **186** |
| 7)  VIP | 1 | U=(乳腺癌+乳癌+乳腺恶性肿瘤)*(水肿+上肢淋巴水肿+淋巴水肿+肿胀)*(临床观察+随机+临床研究+对照组+实验组+治疗组)*(针灸+针刺+艾灸+穴位+针+灸+穴位注射+电针+体针) | **16** |
| 8)  CBM | 1 | (("针灸"[常用字段:智能] OR "针刺"[常用字段:智能] OR "艾灸"[常用字段:智能] OR "穴位"[常用字段:智能] OR "针"[常用字段:智能] OR "灸"[常用字段:智能] OR "体针"[常用字段:智能]) AND ("水肿"[常用字段:智能] OR "肿胀"[常用字段:智能] OR "淋巴水肿"[常用字段:智能] OR "上肢淋巴水肿"[常用字段:智能]) AND ("乳腺癌"[常用字段:智能] OR "乳癌"[常用字段:智能] OR "乳腺恶性肿瘤"[常用字段:智能])) AND ("临床研究"[常用字段:智能] OR "临床观察"[常用字段:智能] OR "随机"[常用字段:智能] OR "对照组"[常用字段:智能] OR "治疗组"[常用字段:智能] OR "实验组"[常用字段:智能]) | **128** |
